# Enhanced High Performance Power Compensation Methodology by IPFC Using PIGBT-IDVR

**DOI:** 10.1155/2015/634846

**Published:** 2015-11-03

**Authors:** Subramanian Arumugom, Marimuthu Rajaram

**Affiliations:** ^1^A.R. College of Engineering and Technology, Tirunelveli 627423, India; ^2^Anna University, Chennai 600025, India

## Abstract

Currently, power systems are involuntarily controlled without high speed control and are frequently initiated, therefore resulting in a slow process when compared with static electronic devices. Among various power interruptions in power supply systems, voltage dips play a central role in causing disruption. The dynamic voltage restorer (DVR) is a process based on voltage control that compensates for line transients in the distributed system. To overcome these issues and to achieve a higher speed, a new methodology called the Parallel IGBT-Based Interline Dynamic Voltage Restorer (PIGBT-IDVR) method has been proposed, which mainly spotlights the dynamic processing of energy reloads in common dc-linked energy storage with less adaptive transition. The interline power flow controller (IPFC) scheme has been employed to manage the power transmission between the lines and the restorer method for controlling the reactive power in the individual lines. By employing the proposed methodology, the failure of a distributed system has been avoided and provides better performance than the existing methodologies.

## 1. Introduction

Energy demands are growing remarkably high, making it extremely difficult for the power quality to meet energy demand which lead to the load shedding problem and power quality problem [1]. The power quality problem is sensed to be an occurrence of nonstandard voltage, current, or frequency [2, 3] which is described as the variation in voltage, current, and frequency in a power system [4–6]. It also refers to a wide variety of electromagnetic phenomena that characterizes voltage and current at a given time and at a given location in the power system [7–9]. The power quality is essential and necessary for proper operation of industrial process concerning a good protection to the system for well-being and progress for prolonged usage [10, 11]. It is clear that the power quality problems such as voltage sag, swell, harmonic distortion, unbalance, transient, and flicker can have a prominent impact on customer devices to result in malfunctions and loss of production [12].

In modern power systems, the use of electronic equipment has become widespread. This electronic equipment is more sensitive towards loads and is less tolerant of short-term voltage disturbances. In power quality enrichment functions, the product of technology-driven custom power and the service solution are closely related to the equipment devices. Among the devices of custom power, the dynamic voltage restorer (DVR) [13] is an economical device that plays an advanced role in a distribution system for reducing the voltage sag [14].

In remote areas, the transmission of load power is necessary to connect the generation sites with lines where electrical power is transmitted through the lines at different voltage levels (usually at 230 kV or higher). By increasing transmission parameters such as capacity and voltage for long distances, the losses during transmission are greatly reduced. Generally, multiple paths exist for the flow of load power in complex interconnected systems. Additionally, control frequently cannot be initiated due to the slow process; therefore, the devices might wear out compared with the devices in static electronic systems.

To reduce these existing difficulties, FACTS technology is employed to handle more services and to improve the reliability of the system. FACTS technology enables the flow of the corresponding load power through the transmission lines under both emergency and normal conditions. The FACTS controller carries the load closer to its thermal rating. For further performance improvement in the system, the static synchronous series compensator (SSSC) has been extended to the interline power flow controller (IPFC).

**Figure 1:**
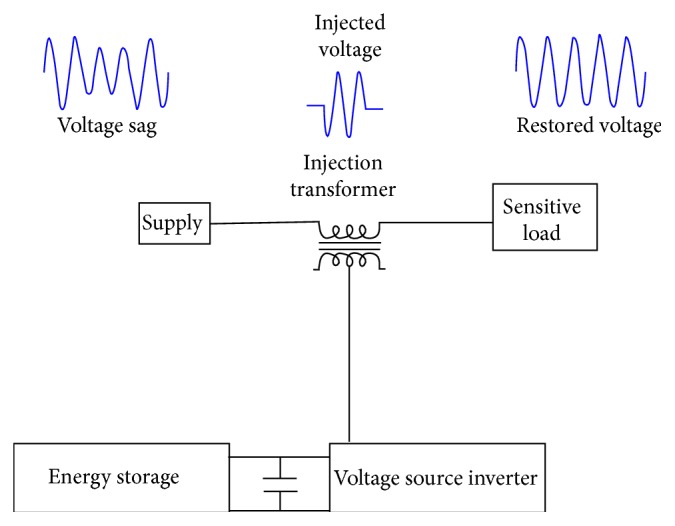
DVR operating principle.

Among various power interruptions, voltage dips create higher-level disruption in the power supply systems. In public distributed systems or in installations, faults occur mainly due to random events and unpredictable voltage dips. By injecting a voltage at the supply mains of distribution in the transient line, the voltage issues could easily be solved. The occurrence of voltage deviation could be compensated by inserting the voltage (phase and magnitude) for upstream distribution. The restorer determines the magnitude and energy of the voltage that is injected by the DVR [15]. The general operating principle of the DVR is schematically displayed in Figure 1.

To reload the energy, interline DVR (IDVR) provides a dynamic energy storage mechanism in the dc-link [16, 17]. IDVR constitutes load sensitive protection from various substations for origination of feeder distribution and thereby shares a common dc-link. The interline power flow controller (IPFC) addresses the common substation issues of transmission lines [18]. The IPFC offers fixed real power transmission capability between the compensated lines, while the reactive power in the lines is adjustable. The IDVR scheme delivers real power transfer through the common dc-link, similar to the IPFC between line loads. However, the lines are originated in IPFC from the substation, whereas in the IDVR system, this happens from various substations. The voltage sag imports real power into the IDVR system from the dc-link, and the mechanism reloads the energy at a specific level to maintain the necessary voltage in the dc-link.

The Parallel IGBT-Based Interline Dynamic Voltage Restorer (PIGBT-IDVR) has been proposed to provide better performance in controlling the power flow and power management. The PIGBT-IDVR embraces the Insulated Gate Bipolar Transistor parallel connection to offer dynamic energy reloading with a lower transition for energy storage in the common dc-link. The IDVR system serves as a starting point for protection of sensitive loads in the distribution feeders from various substations, thereby sharing a common dc-link. The interline power flow controller (IPFC) has been suggested to address the issues related to the transmission lines and to provide real power capability for direct transfer between the compensated lines. The lines are further managed and controlled for reactive power within the system.

The rest of this paper has been organized as follows. A literature review with necessary details on IPFC and IDVR with the proposed PIGBT method is elaborated in Section 2. The proposed work constituting the system design and implementation has been summarized in Section 3.  Section 4 elaborates the simulation results of the proposed model with the necessary discussion. Finally, the last section shows the conclusions from the research work with a few suggestions towards future work.

## 2. Related Work

The dynamic voltage restorer (DVR) delivers an economic and technically advanced solution for voltage sag issue [14, 16]. In distribution systems, the process of voltage restoration involves the injection of real power, the topology of the DVR, long duration compensation for voltage sags, and storage of energy in a dc-link. The interline DVR (IDVR) provides a dynamically efficient method to reload the energy storage in the dc-link. In power systems, various feeders are connected to share the energy storage. Closed-loop load voltage and current-mode control techniques are the two modes of operation. Therefore, the IDVR system provides effectiveness to improve the quality of the power system [19]. In real power flow control mode, based on the requirements of the real power, the performance of the voltage restoration will be processed. There is a clear view of the system mitigation by appearances of lines for long duration for approximately 40% of the voltage sag.

**Figure 2:**
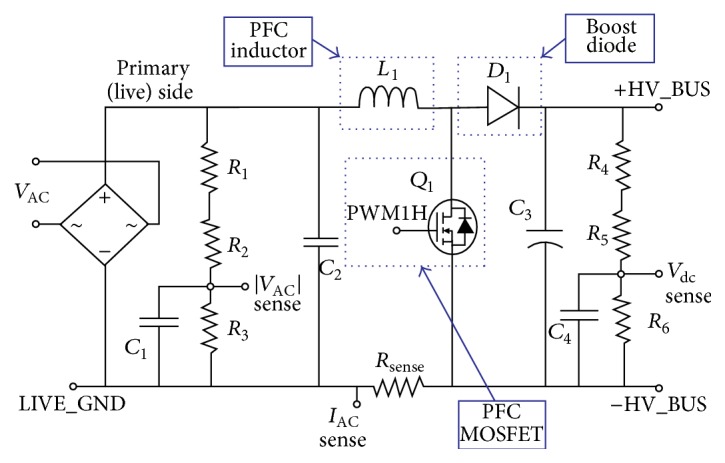
Architectural design of the IPFC system.

The interline power flow controller (IPFC) is based on the Flexible AC Transmission System (FACTS) controller for the voltage source converter (VSC) and for power flow management among the multiline substation transmission system [20]. The power balancing is attained by IPFC through the lines, thereby permitting additional power and improving the power quality. By IPFC circuit modeling, the transfer of real power and voltages is improved further [21]. As an inverter has been employed in the dynamic voltage restorer (DVR), the transformer requirement is reduced and consequently the switching levels are reduced. Ultimately, compared with the DVR topologies, the multilevel inverter utilizes fewer switches and incurs fewer losses at a reduced cost because no injection is needed for the transformers. The switching and control strategies are the compensation strategies for flickers, voltage sags, and swells [15]. The general circuit description of the IPFC system is outlined in Figure 2.

An IGBT-based power stage amplifier provides a valid solution and faster loop-level processing in control systems. The corrected values are chosen to drive and influence the blanking times, for switching and saturation of semiconductors. The correction has been supplied directly to the voltage output of the servo amplifier as a feed forward signal. It consumes substantial power and serves as an alternative approach for complex stage power modeling. Though the design process and the speed of the system are improved, the associated losses in switching have increased and result in increased electromagnetic interference, making the system bulkier and highly expensive. For estimating the voltage in the sensor drive system, the maximum possible accuracy has been found to be limited to the prerequisite value.

The FACT device UPFC has been employed for the control of transmission power flow in the system [17, 22]. With increasing demand for electricity, setting up a new line is not easily possible. The Flexible AC Transmission System (FACTS) utilizes the existing transmission network and a Unified Power Flow Controller (UPFC) using thyristor-controlled devices to emphasize the balancing of voltage power to control the real and reactive power. The control of the reactive power flow in the dc-link is achieved by the shunt converter, and the transmission line and voltage bus of the UPFC are controlled by the series convertor. The shunt converter provides reactive power if the bus voltage of the UPFC is constant during the flow of power. Both the receiving and sending ends are controlled by the bus voltage.

In distributed systems, the quality of power is a major area of concern due to the connection of sophisticated loads. In the industrial environment, voltage sag results in malfunctioning of the equipment. To overcome this issue, a custom power device is connected with the network to improve the voltage disturbances in the electrical system [23]. To control the restorer effectively and dynamically, PI controller and discrete PWM generator have been employed for effective performance analysis under various fault conditions. Use of the PI controller and discrete PWM generator reduces the voltage sags in the DVR [14, 19], becomes efficient and fast in the compensation of the voltage sag, and improves the power quality of the device. The power compensation is found to have achieved approximately 91% with voltage compensation approximately 44%, where an adaptive PI fuzzy controller has been employed for the compensation process of a voltage restorer.

For voltage stability control and compensation of the reactive power, the static synchronous compensator (STATCOM) has been implemented. The STATCOM operates on the principle of IGBT-based VSC. Using the Genetic Algorithm (GA) technique, the capacitor values of the dc-link and the source of the battery have been optimized under diverse load conditions. An interline power flow controller (IPFC) has been employed to control the power flow in individual transmission lines among the multiline transmissions. The IPFC transfers the power in a dc-link using two or more voltage source converters (VSCs) and has the capability of exchanging reactive power in the transmission system.

The deviation in voltage sags results in substantial losses and disruptions. The DVR directly protects voltage sags/swells affecting the sensitive loads on the feeder distribution system. The DVR injects voltage into the lines and thereby maintains the optimum value of the voltage load. The IDVR handles the connection of the restorer with the feeder and shares the storage of energy [24]. DSTATCOM helps to improve the quality of power in the distribution systems, and the voltage sag is compensated. A neural network methodology has been implemented to control and achieve the optimum alleviation of the voltage swell, the voltage sags, and the voltage imbalance. The multilayer perception in neural networks identifies the dynamic sensitive load voltage and thereby regulates this voltage with lower harmonic distortion and faster response [23]. In this paper, the proposed model has been designed to provide better performance for voltage swell, voltage sags, and voltage imbalance. Moreover, the model consists of parallel combinations of IGBT and IDVR to improve the voltage, power, and time requirement for processing, in comparison with the existing methodology.

## 3. The Proposed Scheme

In this section, the proposed system is discussed with its features, analysis, and design methodology. Several changes and losses might occur during power distribution. Therefore, the distribution of the load is processed dynamically as a time-varying phenomenon in these transmission lines. To overcome the shortcomings of the existing systems in order to reduce transmission time and losses, the proposed methodology has been formulated. A distinguished methodology, the Parallel IGBT-Based Interline Dynamic Voltage Restorer (PIGBT-IDVR), has been proposed to achieve power flow control and voltage control for reducing the transmission time and switching time for power from one transmission process to another process.

Here, the functions of the voltage restorer determine the range of compensation, and the inductance is assumed to be negligible. The voltage sags and swells (VSS) function and the injected voltage are expressed as

(1) VSS=VX,n∓VY,nVX,n,VX,n=VY,n∓VI,n.

The maximum possible magnitude of the output voltage is approximately equal to the dc voltage in the various cells of a multilevel inverter. The maximum possible output voltage is expressed by considering (*n* − 1)/2 cells. The derivative of the injected voltage and the maximum value of VSS can be expressed as

(2) VI,n=n−12·Vdi,VSSmax⁡=n−12·VdiVX,n,

where *V*_*X*,*n*_ represents the peak value of the load voltage and the values are potentially equal to a constant value (1 pu). *V*_*Y*,*n*_ represents the peak value of the voltage source, *V*_di_ represents the input of the digital voltage, and the injected voltage is represented by *V*_*I*,*n*_. The level of the output voltage (odd values that are greater than or equal to 3) is represented as *n*.

**Figure 3:**
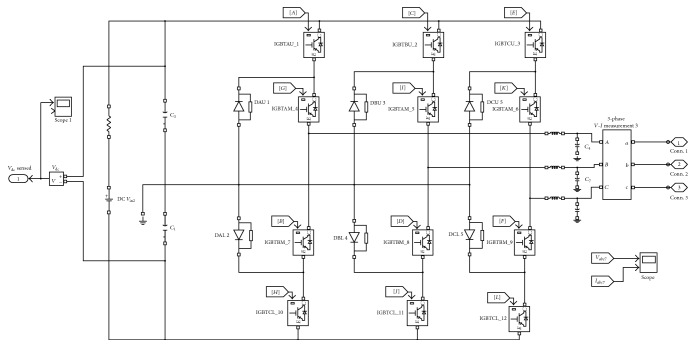
Proposed PIGBT Simulink model.

**Figure 4:**
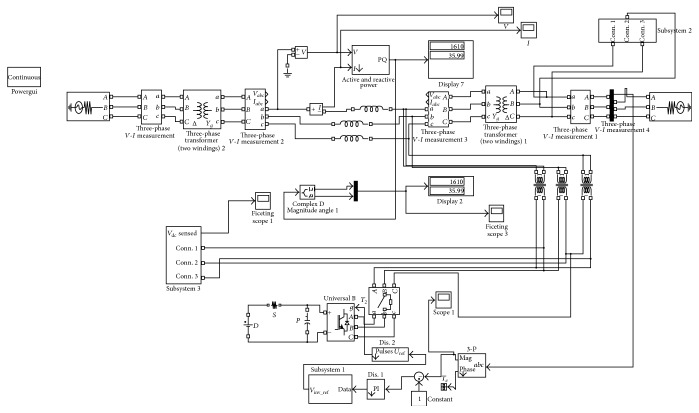
Proposed PIGBT-IDVR Simulink model.

When the modules are connected in parallel, the capability of the current is defined by the parameters that have been used by the individual modules. Due to the variation of parameters between the various modules, the connection impedance that is matching may not provide a realistic sharing of the current. Additionally, an unequal device cooling effect results in a current imbalance within the modules or between the modules. The temperature during switching is directly dependent on the on-state of the respective modules. The dynamic and static current sharing and the current imbalance between the parallel connection modules result in a momentous variation in temperature.  Figure 3 represents the proposed PIGBT Simulink model, and Figure 4 represents the Simulink model for the proposed PIGBT-based IDVR.

**Figure 5:**
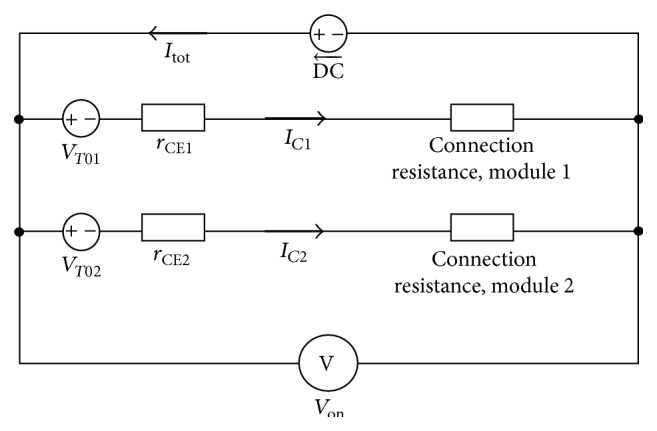
Module representing on-state and current sharing.

**Figure 6:**
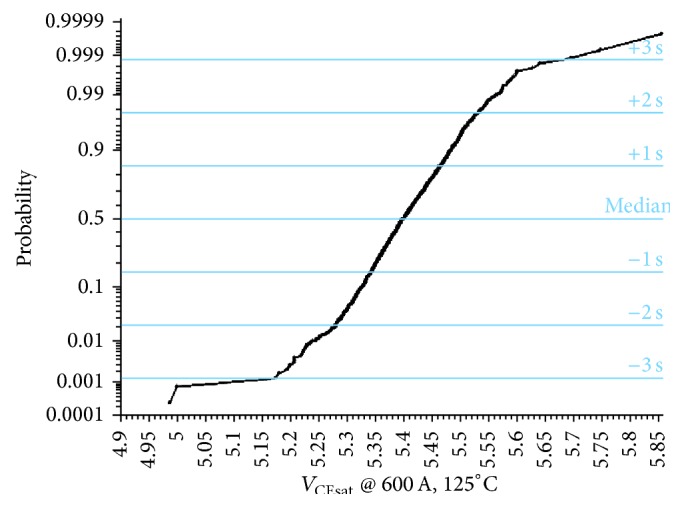
On-state process (6500 V/600 A) module.

**Figure 7:**
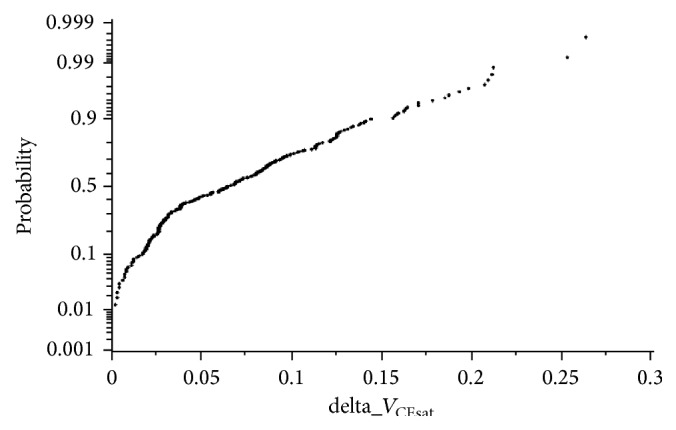
Differences in probability of *V*_CEsat_.

The on-state module and current sharing are influenced by various parallel connection resistances.  Figure 5 shows the parallel connection module of an on-state linear process circuit, and every individual connection contains a resistor. The on-state process module for a particular probability is depicted in Figure 6. The population of median *V*_CEsat_ and deviation are 5.4 V and 0.065 V, respectively, and the maximum difference is 265 mV. An evaluation of the sharing of the module is carried out by grouping the measured module of 2000 into 100 pairs.  Figure 7 clearly displays the differences in probability of *V*_CEsat_.

The voltage difference in the parallel-connected modules shows clearly that current imbalances occur between the modules. To obtain the current imbalance in the modules, an estimate of linear *V*_CEsat_ versus *I*_*c*_ varying between the expected 1/3 of the nominal current and the minimal current (600 A) has been considered. At zero amps, the on-state threshold voltage (*V*_OT_) is found to be 2.5 V:

(3) $$\begin{array}{c} V_{CEsat}\left(\;I_{c}\;\right)=V_{\text{OT}}+I_{c}\cdot r_{\text{os}}. \end{array}$$

The imbalance of current has to be evaluated because of variations in the parallel-connected module, which shows the same drop in voltage, assuming connection resistance to be zero. The results of the module current have been estimated on the basis of average *V*_CEsat_ and also on its on-resistance (*r*_os_). The current imbalance in the proposed module is functionally evaluated as

(4) $$\begin{array}{c} I_{c\left(n\right)}=\frac{\sum_{n=0}^{m}V_{\text{CEsat}\left(n\right)}/2-V_{\text{OT}\left(n\right)}}{r_{\text{os}\left(n\right)}}. \end{array}$$

**Figure 8:**
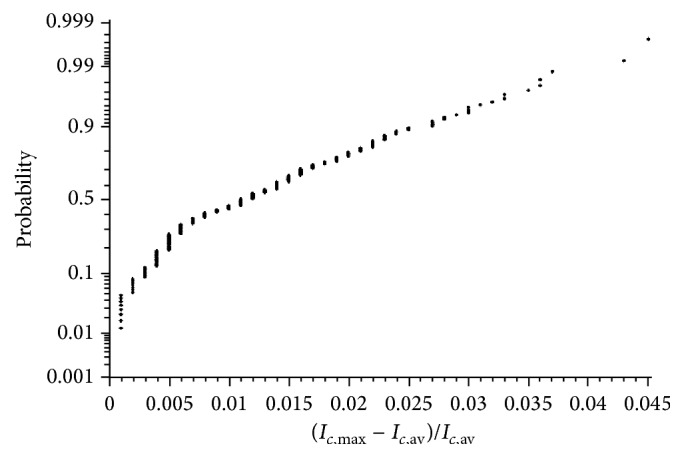
Current imbalance.

The probability of current imbalance of the parallel-connected module has been obtained as depicted in Figure 8. The current imbalance that has been evaluated is found to have a median of 1.02% and a maximum of 4.5%.

By the on/off switching behavior of the inverter, the losses and blanking times have been avoided to the maximum extent. It provides a nonlinear process in the power stages, and the switching voltage during emerged time sequences has been processed on the basis of the switching level and the flow of current.

**Figure 9:**
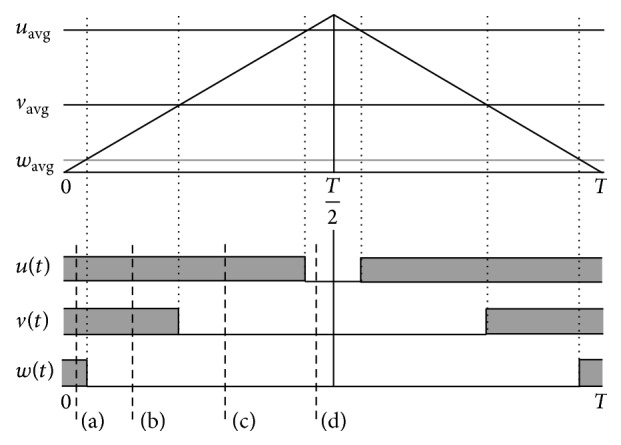
General structure of sequence generation.

**Figure 10:**
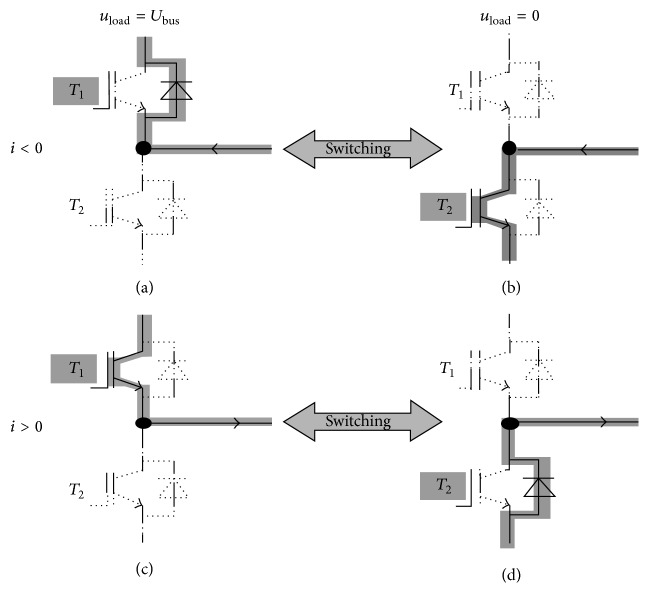
On-state switching and current flow in power stage.

The on-time evaluation is necessary in representing the state and signal of switching from one transmission line to another. The actual times and preceding times of the carrier signal are found to be triangular in shape as seen from Figure 9. Proper sequence of switching should be assigned for attaining minimum current harmonics and current losses. Each switch is considered to operate on a complete cycle (both on and off switching).  Figure 10 shows the inverter state of switching, and the cycle begins and ends with the zero vector, with the same voltage at all of the terminals. Additionally, the general flow of switching and on-state current can be understood from the figure below. The current/voltage will be changed even if the state of switching remains unchanged; thus, the switches are forced to assume dissimilar states with time.

In the parallel connection of modules, the connection derating is based on Safe-Operating-Area (SOA) and thermal derating. SOA cares for the current imbalance and the switching flow. It was found that 50% of the delay in switching occurs in the off mode. Therefore, the maximum turn-off in the process has been reduced in the SOA itself. In thermal derating, the current sharing in a homogeneous process leads to higher losses only in the parallel-connected module. The losses in on-state are expressed with the factor of current imbalance due to the current mismatch of 5% having *D* = 1.05.

The losses in the switching mismatch in parallel connection operating at the maximum temperature are expressed as

(5) $$\begin{array}{c} P_{\text{ST}}=\left(\;V_{\text{OT}}+r_{\text{CE}}\cdot I_{c}\;\right)\cdot I_{c}\cdot D. \end{array}$$

The strategy of the proposed control system circuit has been expressed with its function as per the circuit in Figure 4, and the following expressions have been obtained:

(6) Ic=CfdVidT,VX=VY+VI,Vo=VI+rfjf+XfdjfdT,jf=jX+jI.

The expression for injected voltage *V*_*I*_(*S*), with the transform function and using the above equations, is defined as

(7) VIS=1XfIfs2+rfIfS+1VIS+rf+LfSXfIfs2+XfIfS+1CX.

## 4. Simulation Study and Performance Analysis

In this section, the simulation results of the proposed system with necessary analysis have been worked out. These evaluations are carried out to verify the compensator requirements of the proposed PIGBT-IDVR system.

**Figure 11:**
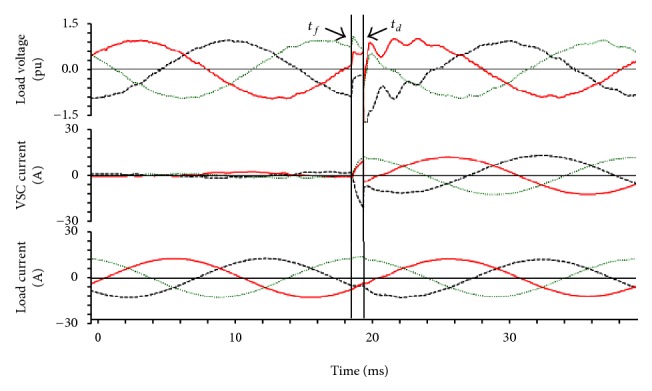
Simulation results of VSS with single phase.

Figure 11 clearly shows that delay occurs in the process only if there is any disconnection or if any other issues occur. The VSC current is almost zero till 20 msec. After 20 msec, the current rises correspondingly and rapidly. The load voltage shows a linear variation till 20 msec, but after that particular point the linearity becomes disturbed. However, the load current shows a linear variation from the beginning until the end.

**Figure 12:**
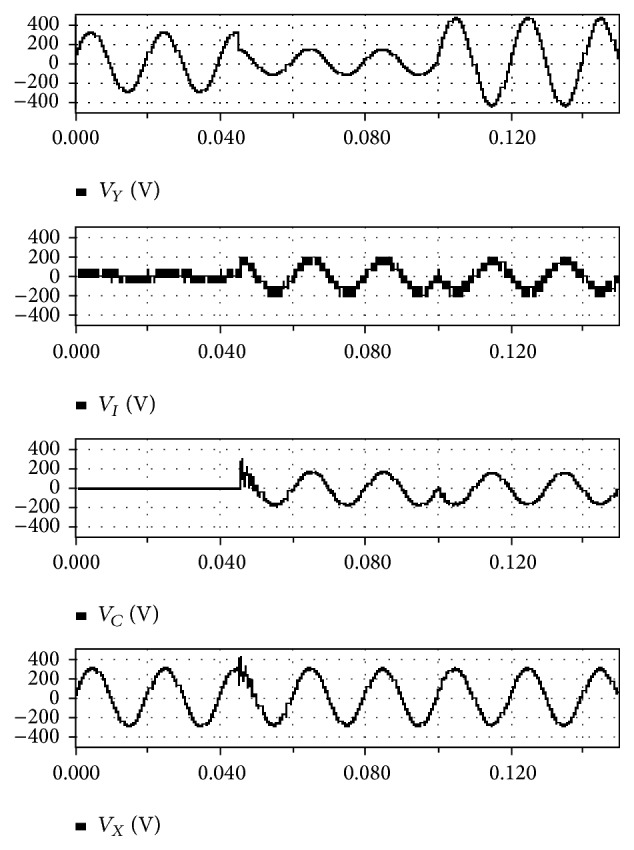
VSS compensation.

Figure 12 depicts the voltage sag and swells compensation. Here, *V*_*Y*_ (V) is the source voltage of the proposed module, *V*_*I*_ (V) is the voltage output, *V*_*C*_ (V) is the compensated and injected load voltage, and finally *V*_*X*_ (V) is the load voltage of the system. The figure clearly shows that the source voltage drops to a minimum value at the center of the cycle, but the source voltage shows a linear and maximum value at the beginning and the end of the cycle.

The voltage output of the proposed methodology shows a linear variation only after some predetermined value of the time. Additionally, the compensated and injected load voltages show a linear variation only after some predetermined value of time. The load voltage of the proposed system shows a linear variation from the beginning to the end of the cycle, clearly indicating that the proposed methodology produces a nonfluctuating, nonvarying, and linear voltage at the output, attained mainly by the employment of novel methods in the proposed system. This output voltage could be effectively employed for controlling the system components and thus the proposed methodology could be employed for high performance and high speed dynamic control systems.

**Figure 13:**
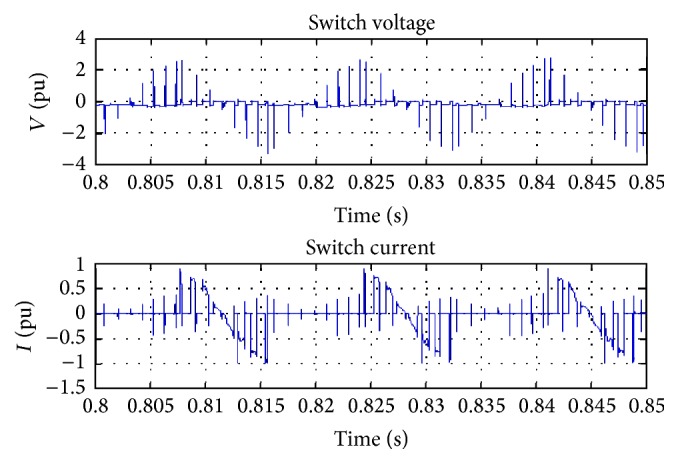
Switching of voltage and current.

The voltage switching and current switching are carried out on the basis of the module functionality, and the switching process is depicted in Figure 13. The figure clearly indicates that the switching of voltage and current happens in an alternative manner. The performances are better compared with the existing systems.

**Figure 14:**
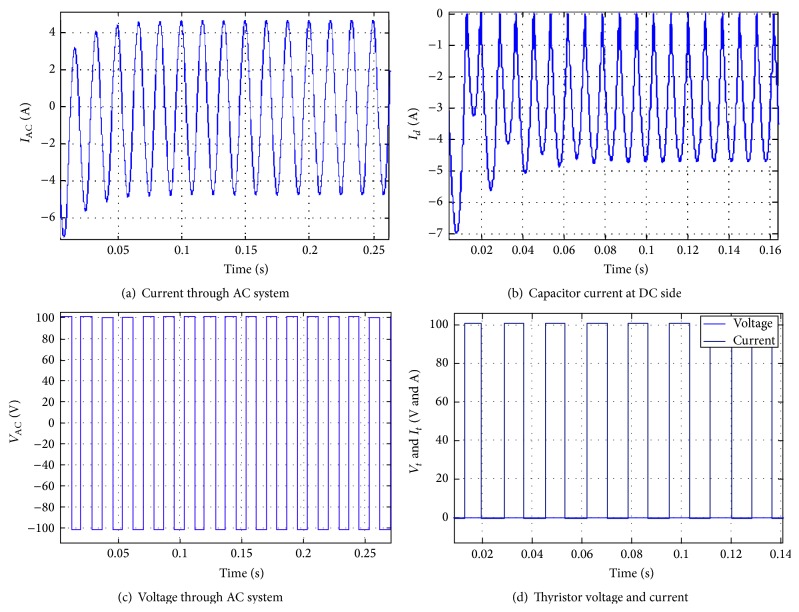
Simulation results of VSC single phase with constant voltage source (DC and AC supply).

**Figure 15:**
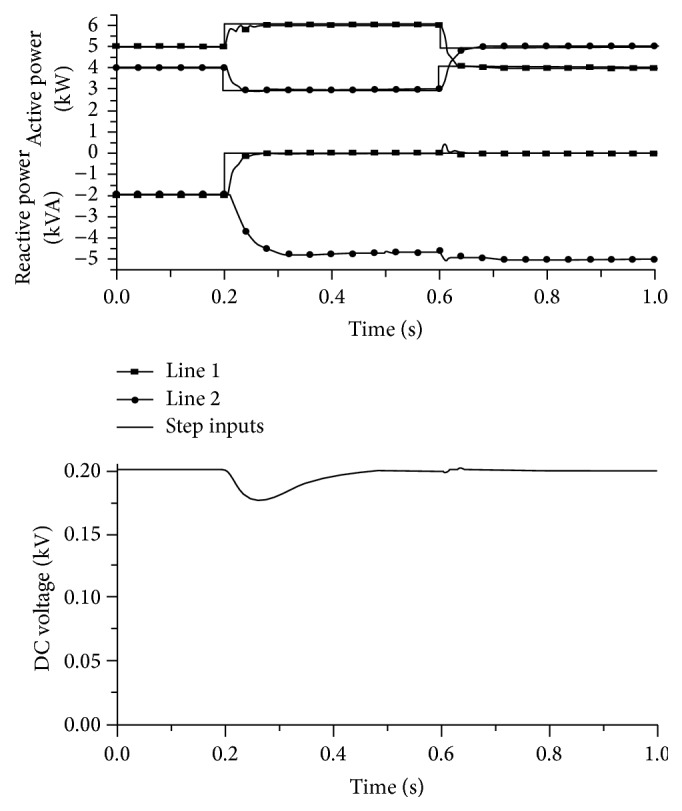
Simulation results of various transmission parameters (double line).

Figure 14 shows the simulation results of VSC with a constant voltage source of DC and AC supplies. Figures 14(a) and 14(b) show the current flow in an AC system and the current of the capacitor in the VSC, respectively. Additionally, Figures 14(c) and 14(d) represent the voltage of the VSC and the VSC single phase, respectively. The current flow in the AC system and the current through the capacitor are found to be linear-valued, after some predetermined time value. The voltages of the VSC show a linear and maximum value, thereby clearly indicating that the proposed methodology could be used for high speed switching applications. The corresponding voltage and current transmission in a line are represented in Figure 15.

**Figure 16:**
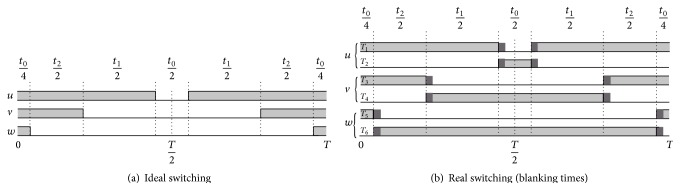
Switching state and effects of blanking times.

**Figure 17:**
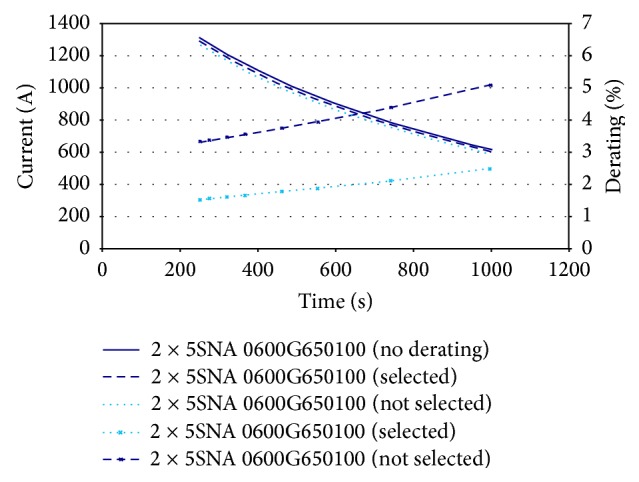
Derating current output.

Figure 16 shows the effects of the blanking time of the switching state. Correspondingly, the derating current output is shown in Figure 17. The derating current maintains an almost gradual increase, as represented by the lowest line in the graph. The current is also found to be the minimum value, as desired.

**Figure 18:**
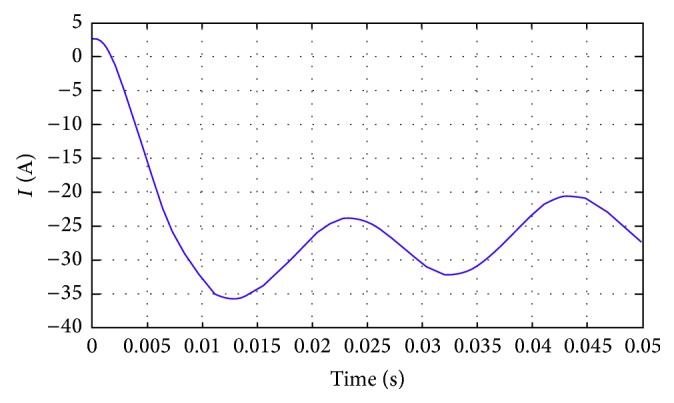
Current flow in the line.

**Figure 19:**
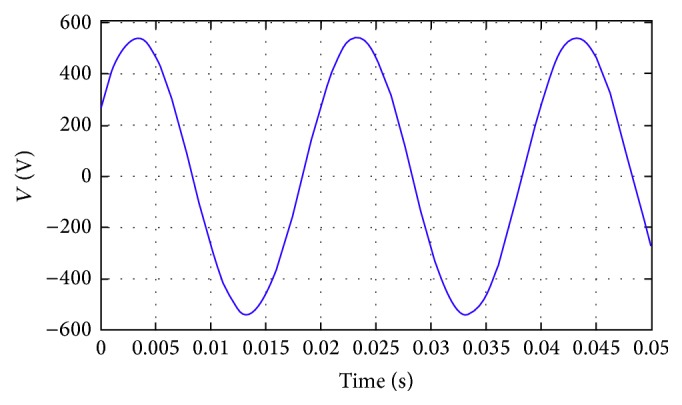
The voltage in the line.

Figures 18 and 19 show the current flow and voltage in a line for the proposed methodology. The current attains a steady value after only 0.015 seconds. Initially, the current flow is almost 5 amperes, but due to the novel methodologies employed in the proposed architecture, the current flow is dropped down to −25 amperes. The linearity is further maintained till the end of the process in the proposed methodology.

**Figure 20:**
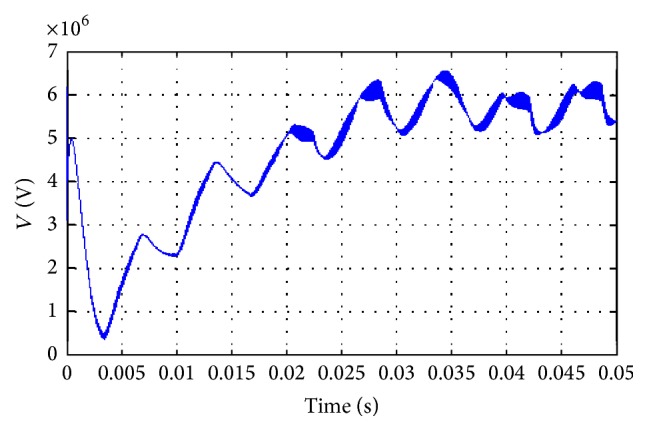
Power compensation for output voltage.

**Figure 21:**
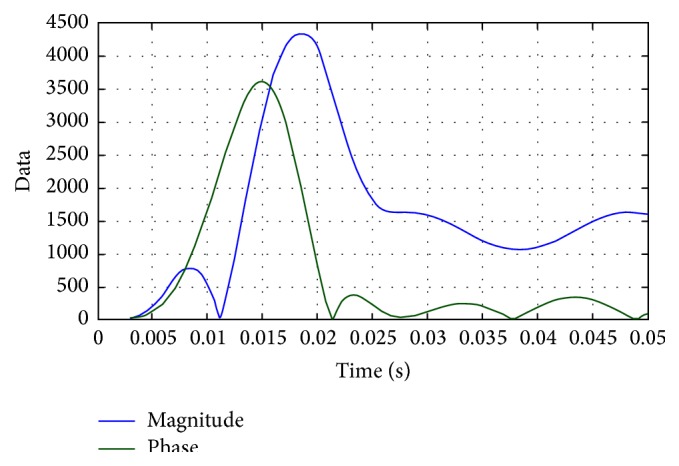
Magnitude and phase of power compensation.

The power consumption and the magnitude and phase of the power are represented in Figures 20 and 21, respectively. From the beginning of the process to 0.015 seconds, the power compensation is very low for the proposed methodology. The power compensation further increases slowly. The power compensation sees a steady value only after 0.025 seconds. This steady value is further maintained until the end of the process, clearly indicating that the proposed system provides better compensation compared with the existing methodologies. Further, in the proposed methodology, the compensation could be attained with proper magnitude and phase value.

## 5. Conclusion and Future Work

In this paper, a new methodology called the Parallel IGBT-Based Interline Dynamic Voltage Restorer (PIGBT-IDVR) method has been proposed, which mainly targets the dynamic processing of energy reloads in common dc-link energy storage with adaptively less transition time. The operating principles of the proposed methodology have been explained clearly. The simulation results of the proposed module provide better compensation than the existing system. The evaluation of the proposed methodology has been performed in a common dc-link to provide a faster process, reduction in switching losses, dynamic processing of energy reload with less transition in energy storage, less transmission time, and proper management in the flow of current and voltage. The simulated results have been well evaluated, and the performance of the proposed PIGBT-IDVR model has been analyzed to prove the effectiveness of the proposed model in the MATLAB/Simulink environment. In future research, the multilevel inverter concept shall be employed for the power of an electronic system with medium operating voltage. For low frequency operation and with less distortion, the multilevel concept will be a better alternative methodology. This research work could be extended in the future to obtain easy process and better performance.
